# Diseases of the Throat

**Published:** 1872-09

**Authors:** 


*Diseases of the Throat.* A Guide to the Diagnosis and Treatment of Affections of the Pharynx, Œsophagus, Trachea, Larynx, and Nares. By J. SOLIS COHEN, M. D. Illustrated. New York: WM. WOOD & Co., 1872. Buffalo: T. Butler & Son.

The treatment of diseases of the throat and air passages for many years has been or rather was the especial aversion of physicians. Lacking many, in fact all, of the modern appliances to aid diagnosis and treatment. With none of the well written monographs and text books of the present time to guide them, the treatment of diseases of the throat had been both unsatisfactory to physicians and without benefit to their patients. Within the last decade rapid strides in advance have been made in this department of medical science. The attention of scientific investigation has been directed to this class of diseases, and the results will compare favorably with any that have been achieved in other fields of labor. New laws have been discovered, and new applications of old ones been made. New instruments and appliances have been invented and the results of observation have been recorded in several very excellent monographs and hand books. Foremost among the students of this class of maladies in this country has been Dr. Cohen, who has already placed the profession under many obligations to him for his well-made and carefully recorded observations. The need of a complete text-book by an American author for American physicians has long been felt, and the appearance of Dr. Cohen's work will be hailed will feelings of delight by the profession, which we are sure will not be disappointed by careful and close observation. The work is divided into fifteen chapters, with extensive references upon subjects treated on in the text. The first seven chapters treat of diseases and affections of the throat, the remainder of the work is devoted to affections of the tonsils, palate and uvula, pharynx, œsophagus, nasal passages, frontal sinus, larynx and trachea, and to diseases of the neck affecting the deeper tissues of the throat secondarily. Dr. Cohen treats his subjects in a pleasing and intelligent manner and in language which will be readily understood by the general practitioner as well as by the specialist. The text is in clear plain type, and the illustrations are well made and aptly chosen. The publishers have in their fine style placed a book in the hands of the physician which, while being of much intrinsic value, will be an ornament to his library.

